# Complete chloroplast genome of *Impatiens huangyanensis* Jin and Ding 2002: genomic features and phylogenetic relationship within genus *Impatiens* (Balsaminaceae)

**DOI:** 10.1080/23802359.2023.2280277

**Published:** 2023-11-10

**Authors:** Ming Jiang, Yan Zhu, Junfeng Wang, Honghua Bao, Huijuan Zhang

**Affiliations:** a College of Life Sciences, Taizhou University, Taizhou, China; b Scientific Research Management Center, East China Medicinal Botanical Garden, Lishui, China; c Luqiao Branch, Taizhou Municipal Ecology and Environment Bureau, Luqiao, China

**Keywords:** *Impatiens huangyanensis*, characterization, chloroplast genome, phylogenetic analysis

## Abstract

*Impatiens huangyanensis* Jin and Ding 2002 is a plant species with very small populations, and it distributes only in Huangyan, Zhejiang Province, China. In this study, the complete chloroplast genome of *I. huangyanensis* was assembled by using high-throughput Illumina paired-end sequences. Its genomic feature was determined, and comparative genomic analysis of the genus *Impatiens* was performed. The results revealed that the full-length chloroplast genome of *I. huangyanensis* was 152,156 bp with a GC content of 36.8%. The chloroplast genome contains a typical quadripartite structure, comprising two copies of inverted repeats (IRs), a small single-copy (SSC) region, and a large single-copy (LSC) region. The sequence lengths of IR, SSC, and LSC were 25,756 bp, 17,662 bp, and 82,982 bp, respectively. The chloroplast genome consisted of 134 genes, including 84 protein-coding genes, 37 transfer RNA genes, eight ribosomal RNA genes, and five pseudogenes. Phylogenic results indicated *I. huangyanensis* shared a clade with *I. davidii* Franchet 1883, *I. macrovexilla* Chen 2000, *I. fanjingshanica* Chen 1999, and *I. piufanensis* Hook 1908, with a support rate of 100%. Our study provided insight into further studies on the conservation genetics of *I. huangyanensis*.

## Introduction

The Balsaminaceae is a widely distributed family that includes only two genera, namely *Hydrocera* and *Impatiens. Hydrocera* is a monospecific genus, containing only one species *H. triflora*, distributed in tropical Asia such as Southeast Asia, South India, and China (Shui et al. 2011). *Impatiens* is a species-rich genus, comprising over 1,000 species, mostly annual and perennial herbs with succulent stems (Janssens et al. 2006). China is regarded as the center for origin and diversification of Balsaminaceae, and there are about 250 wild *Impatiens* species, many of which have long been utilized as medicinal herbs (Luo et al. 2021). *I. huangyanensis* is a narrowly distributed *Impatiens* species, which was found only in mountain areas of Huangyan, Zhejiang Province, occurring in habitats of roadsides and forest margins, with very small populations (Jin and Ding 2002). At present, the chloroplast genome of *I. huangyanensis* has not been reported, and its systematic genetic location remains unclear. In this study, the chloroplast genome of *I. huangyanensis* was assembled based on high-throughput paired-end sequences, and a phylogenetic tree was generated to reveal its relationship with other *Impatiens* species.

## Materials and methods

### Plant sampling

**Figure 1.**
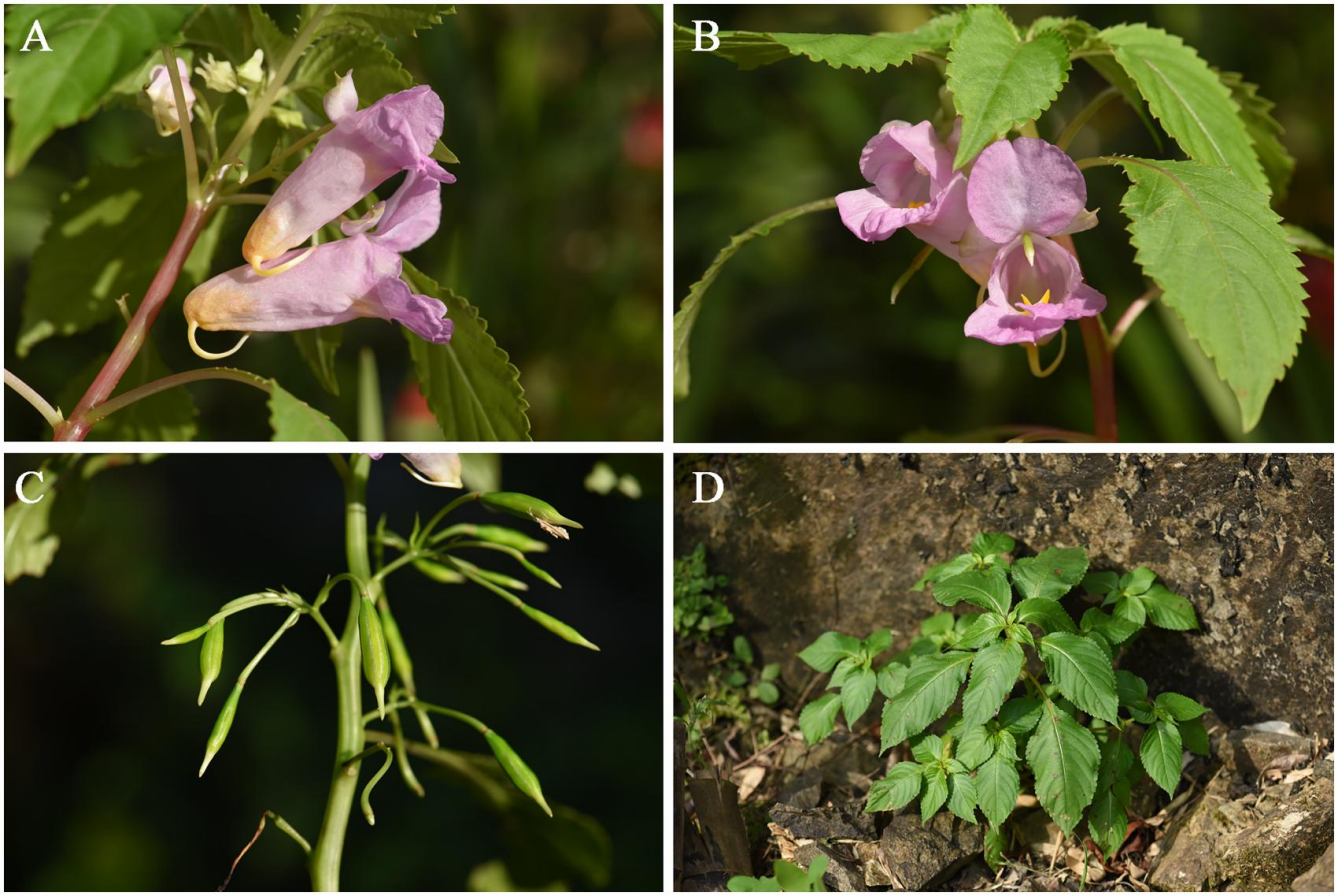
*Impatiens huangyanensis* Jin and Ding 2002. (A) Flower lateral view; (B) flower front view; (C) fruits; (D) natural habitat of *I. huangyanensis*. All the photos were taken by Ming Jiang.

Fresh leaves were gathered from Foling (28°32′25″ N, 121°09′37″ E), Huangyan, Zhejiang Province, China (Figure 1). Leaves were taken to the laboratory and then washed with running water to get rid of dirt and dust before rinsed with sterile distilled water. A voucher specimen namely CHS20200388 was deposited in the Molecular Biology Innovation Laboratory at Taizhou University (Dr. Ming Jiang, jiangming1973@139.com).

### DNA isolation, sequencing, assembling, and annotation of the chloroplast genome

The sodium dodecyl sulfate method was applied to extract high-quality genomic DNA. The genomic DNA was used to construct paired-end sequencing libraries with an average insert size of 350 bp. The library was sequenced by an Illumina Hiseq X Ten sequencing platform. Low-quality reads were filtered by using NGSQCToolkit v2.3.3 (Patel and Jain 2012). Chloroplast genome assembly was performed using the NOVOPlasty program (Dierckxsens et al. 2017). The chloroplast genome was annotated by Dual Organellar GenoMe Annotator (Wyman et al. 2004). Transfer RNA gene prediction was conducted by tRNAscan-SE 2.0.9 (Chan and Lowe 2019). The whole chloroplast genome map of *I. huangyanensis* was drawn using CPGView (http://www.1kmpg.cn/cpgview/; Liu et al. 2023). Sliding window analysis of nucleotide diversity was performed by using DnaSP 6.0 (Rozas et al. 2017).

### Phylogenetic analysis

To understand its relationship with other *Impatiens* species, 18 chloroplast genome sequences of *Impatiens* species were downloaded from GenBank (National Center for Biotechnology Information [NCBI]) to construct a phylogenetic tree. We also downloaded a sequence of *H. triflora* (L.) Wight. et Arn. 1753 (NCBI accession number: NC_037400), which was used as an outgroup species. The chloroplast genomes were aligned with MAFFT v7.450, a multiple sequence alignment program (Katoh and Standley 2013). Based on the best model GTR + R, a phylogenetic tree was built by PhyML 3.1 with the maximum-likelihood method using whole chloroplast genome sequences (Guindon et al. 2010).

## Results

**Figure 2.**
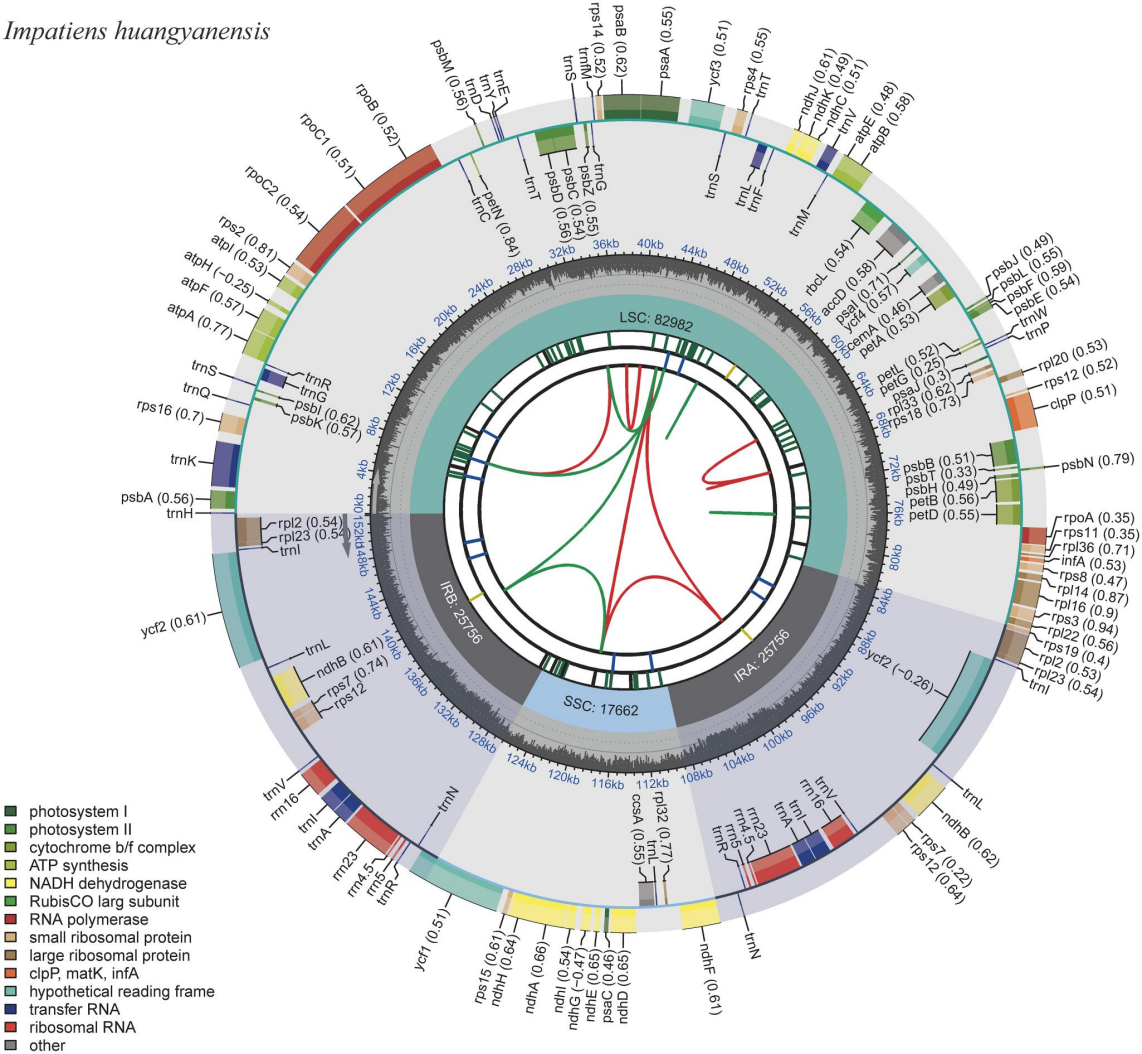
The chloroplast genome of *Impatiens huangyanensis*. The map contains six tracks. From the center outward, the first track shows the dispersed repeats, which consist of direct and palindromic repeats, connected with red and green arcs. The second track indicates the long tandem repeats as short blue bars. The third track reveals the short tandem repeats or microsatellite sequences as short bars with different colors. The colors, type of repeat they represent, and the description of the repeat types are as follows: black: c (complex repeat); green: p1 (repeat unit size = 1); yellow: p2 (repeat unit size = 2); purple: p3 (repeat unit size = 3); blue: p4 (repeat unit size = 4); orange: p5 (repeat unit size = 5); red: p6 (repeat unit size = 6). the chloroplast genome contains an LSC region, an SSC region, and two IR regions, and they are shown on the fourth track. The GC content along the genome is shown on the fifth track. Genes are color-coded according to their functional classification. The transcription directions for the inner and outer genes are clockwise and anticlockwise, respectively. The bottom left corner indicates the key for the functional classification of the genes.

Totally, 3.27G clean data were obtained, with 10,886,063 reads. The results revealed the complete chloroplast genome of *I. huangyanensis* was 152,156 bp in length, with an average depth of 3164.80× (Supplementary Figure S1). Among the *Impatiens* chloroplast genomes used in this study, the sequence lengths ranged from 151,538 bp (*I. fanjingshanica* Chen 1999) to 152,928 bp (*I. mengtszeana* Hooker 1908). The chloroplast genome consisted of four regions, a large single-copy (LSC), a small single-copy (SSC), and two inverted repeats (IRs), and the lengths of LSC, SSC, and IRs were 82,982 bp, 17,662 bp, and 25,756 bp, respectively (Figure 2). The GC content of the plastid genome was 36.8%, which was equal to those of *I. fanjingshanica*, *I. hawkeri* Bull 1887, *I. walleriana* Hooker 1868, *I. glandulifera* Royle 1834, *I. piufanensis* Hook 1908, *I. cyanantha* Hook 1908, *I. macrovexilla* Chen 2000, and *I. uliginosa* Franchet 1886. IRs of *Impatiens* plant species used in this study were not conserved, they varied from 82,542 bp (*I. fanjingshanica*) to 83,741 bp (*I. chlorosepala* Handel-Mazzetti 1934). The plastid genome sequence of *I. huangyanensis* was submitted to GenBank with an accession number of OR139616.

The chloroplast genome consisted of 134 genes, including 84 protein-coding genes, 37 tRNA genes, eight rRNA genes, and five pseudogenes. Eighteen genes including *ndhB*, *rpl23*, *trnA-UGC*, *trnG-GCC*, *trnI-CAU*, *trnI-GAU*, *trnL-CAA*, *trnR-ACG*, *trnV-GAC*, *ycf1*, *ycf2*, *ycf15*, *rpl2*, *rps12*, *rrn4.5*, *rrn5*, *rrn16*, and *rrn23* contained two copies. A total of 13 genes had one or two introns, these genes included *atpF*, *clpP*, *ndhA*, and *ndhB* (two copies), *petB*, *petD*, and *rpl2* (two copies), *rpl16*, *rpoC1*, *rps16*, and *ycf3* (Supplementary Figure S2). The *matK* and both copies of *ycf15* were found to be pseudogenized due to the presence of internal stop codons, while the *ycf1* gene overlapped with *ndhF* on SSC/IR border and rps19 on IR/LSC border were pseudogenized owing to truncations at their 3′ ends. The *rps12* is a trans-splicing gene (Supplementary Figure S3). By using DnaSP, *trnG-GCC*, *ndhF-rpl32*, and *ycf1* were identified as highly variable regions (Supplementary Figure S4).

**Figure 3.**
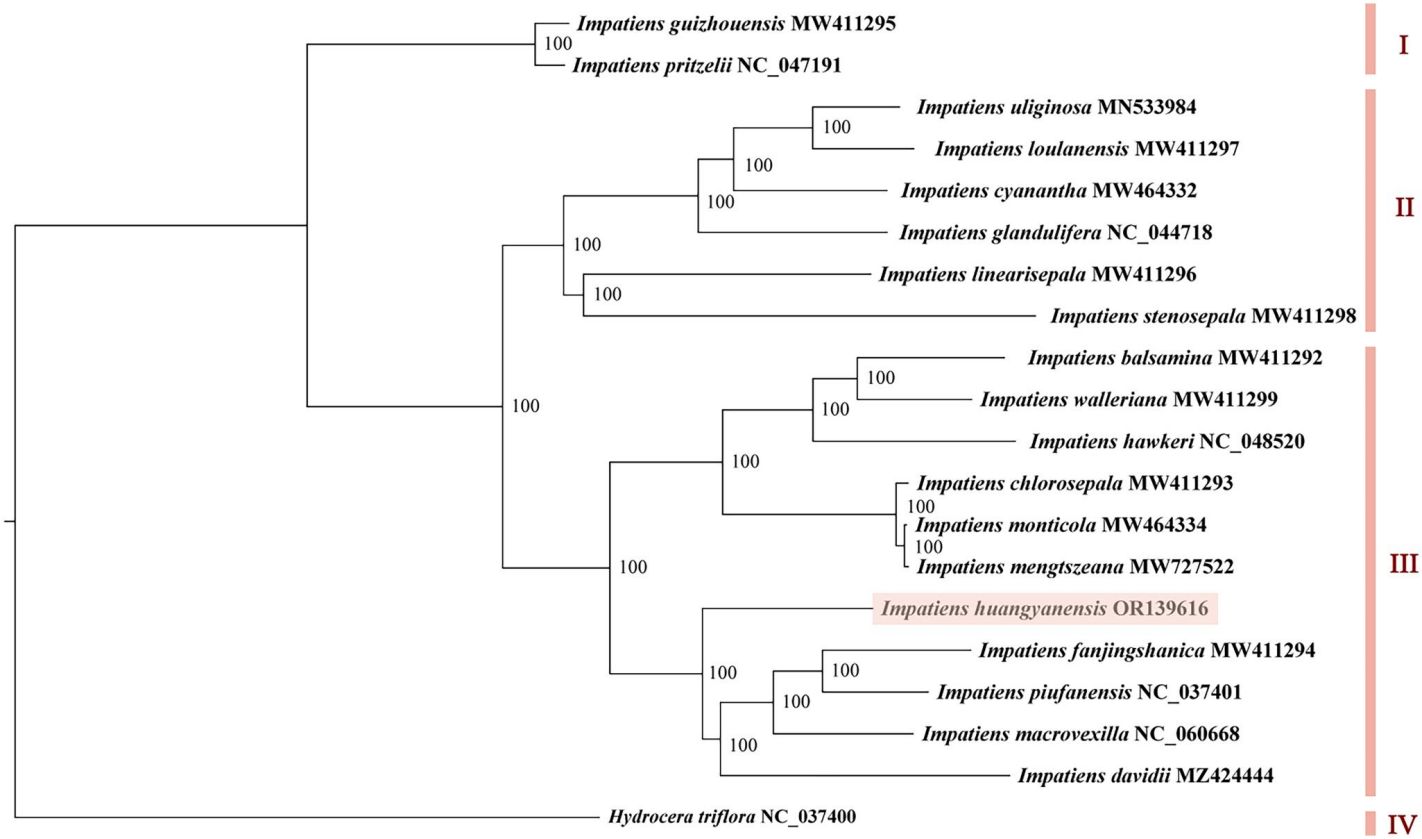
The maximum-likelihood tree based on complete chloroplast genome sequences of *Impatiens huangyanensis* and 18 other *Impatiens* species, with *Hydrocera triflora* as the outgroup species. The numbers next to the nodes show bootstrap support values. The phylogenetic tree was generated by PhyML 3.1 with the maximum-likelihood method.

The phylogenetic analysis results showed that the 20 Balsaminaceae species clustered into four major groups. Both *I. guizhouensis* Chen 1999 and *I. pritzelii* Hook 1908 shared group I, and *I. uliginosa*, *I. loulanensis* Hooker 1911, *I. cyanantha*, *I. glandulifera*, *I. linearisepala* Akiyama Ohba & Wu 1996, and *I. stenosepala* Pritzel 1900 gathered in group II. The outgroup species *H. triflora* was alone in group IV, while the remaining 11 species, including *I. huangyanensis*, were in another group (III). *I. huangyanensis* was sister to *I. davidii* Franchet 1883, *I. macrovexilla*, *I. fanjingshanica*, and *I. piufanensis*, with a support rate of 100% (Figure 3).

## Discussion and conclusions

*Impatiens* is a species-rich genus of angiosperms, and a number of new species were described (Tiwari 2023). However, the chloroplast genomes of most *Impatiens* species have not yet been assembled. In this study, we assembled *I. huangyanensis* chloroplast genome and revealed its close relationship with *I. davidii*, *I. macrovexilla*, and *I. piufanensis*. Pseudogenization is a common phenomenon in chloroplast genome. In our present study, five genes were found to be pseudogenized. The pseudolization of *matK* was also observed in *Anthoceros formosae* Steph. 1916, *Campylotropis bonii* Schindl. 1916, and some photosynthetic orchid species (Kugita et al. 2003; Barthet et al. 2015; Feng et al. 2022). One copy of *ycf1* is located at the boundary of SSC/IR, and its 3′ ends were truncated, pseudogenization of *ycf1* is common due to the incomplete duplication of the normal copy (Amar 2020).

Assembly and phylogenetic analysis of *I. huangyanensis* chloroplast genome sequence provided useful data for further studies on population structure and conservation of *Impatiens* genus.

## Supplementary Material

Supplemental Material

## Data Availability

The data that support the findings of this study are openly available in GenBank of NCBI at https://www.ncbi.nlm.nih.gov/nuccore/ OR139616. The associated BioProject, SRA, and Bio-Sample numbers are PRJNA897866, SRR22178406, and SAMN31582508, respectively.
