# Supplementary material for: Complete chloroplast genome of Impatiens huangyanensis Jin and Ding 2002: genomic features and phylogenetic relationship within genus Impatiens (Balsaminaceae)
Source: Mitochondrial DNA B Resour. 2023 Nov 10;8(11):1229–33. doi: 10.1080/23802359.2023.2280277 (PMC10653657; doi:10.1080/23802359.2023.2280277)
Supplement: Supplemental Material [file TMDN_A_2280277_SM5733.docx]

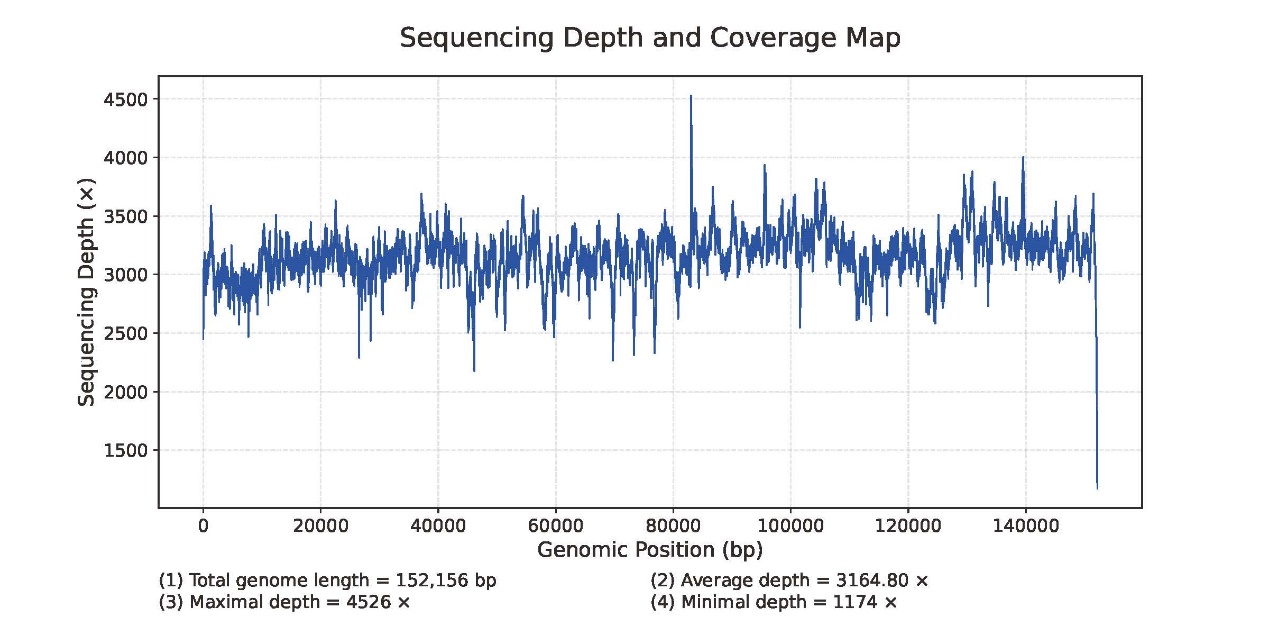


**Figure S1** Coverage depth figure of the *Impatiens huangyanensis* chloroplast genome. The horizontal coordinate is the position of the chloroplast genome, and the vertical coordinate is the sequencing depth.


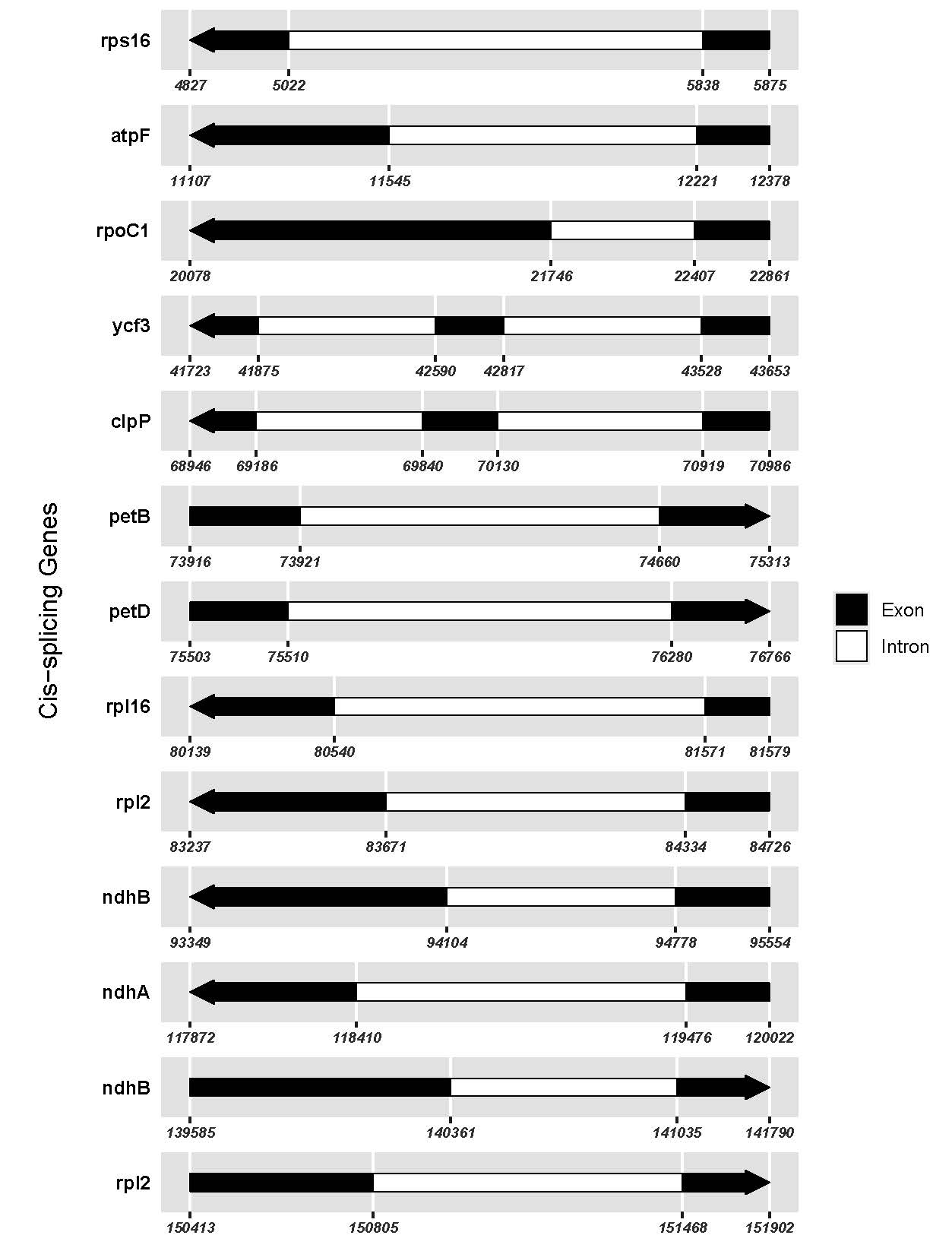


**Figure S2** Schematic map of the cis-splicing genes in the *Impatiens huangyanensis* chloroplast genome.





**Figure S3** Schematic map of the trans-splicing gene *rps12* in *Impatiens huangyanensis* chloroplast genome.


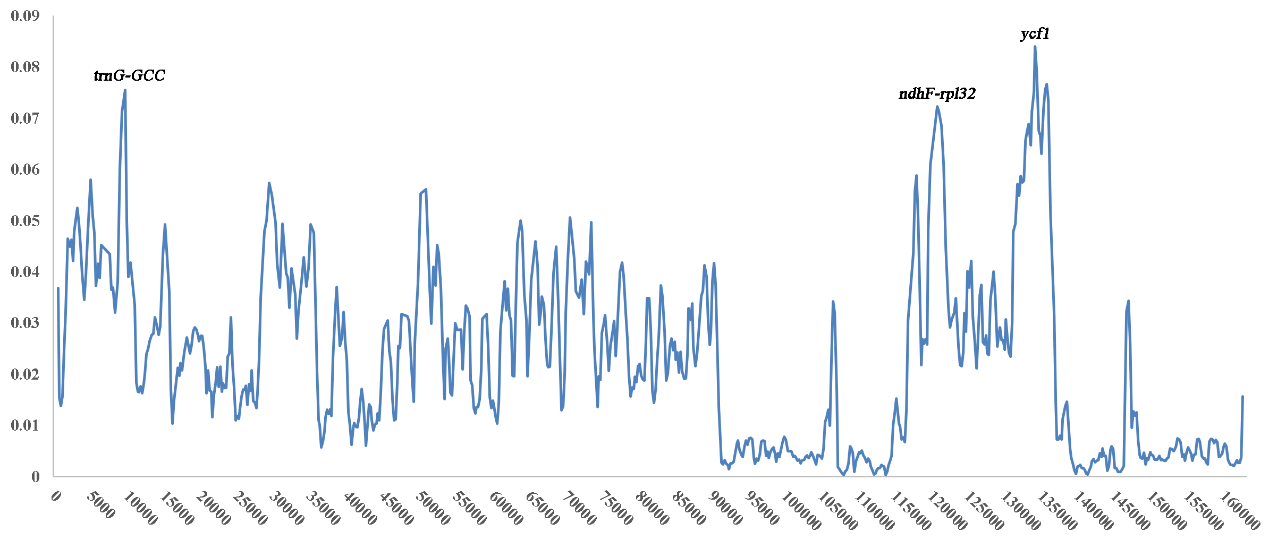


**Figure S4** Sliding window analysis of nucleotide diversity by using DnaSP 6.0. *TrnG-GCC*, *ndhF-rpl32*, and *ycf1* indicate the three highly variable regions.
